# An expansion of rare lineage intestinal microbes characterizes rheumatoid arthritis

**DOI:** 10.1186/s13073-016-0299-7

**Published:** 2016-04-21

**Authors:** Jun Chen, Kerry Wright, John M. Davis, Patricio Jeraldo, Eric V. Marietta, Joseph Murray, Heidi Nelson, Eric L. Matteson, Veena Taneja

**Affiliations:** Department of Health Sciences Research, Division of Biomedical Statistics and Informatics, Mayo Clinic, 200 First St. S.W., Rochester, MN 55905 USA; Department of Medicine, Division of Rheumatology, Mayo Clinic, 200 First St. S.W., Rochester, MN 55905 USA; Department of Gastroenterology, Mayo Clinic, 200 First St. S.W., Rochester, MN 55905 USA; Department of Surgery, Mayo Clinic, 200 First St. S.W., Rochester, MN 55905 USA; Department of Immunology and Division of Rheumatology, Mayo Clinic, 200 First St. S.W., Rochester, MN 55905 USA

## Abstract

**Background:**

The adaptive immune response in rheumatoid arthritis (RA) is influenced by an interaction between host genetics and environment, particularly the host microbiome. Association of the gut microbiota with various diseases has been reported, though the specific components of the microbiota that affect the host response leading to disease remain unknown. However, there is limited information on the role of gut microbiota in RA. In this study we aimed to define a microbial and metabolite profile that could predict disease status. In addition, we aimed to generate a humanized model of arthritis to confirm the RA-associated microbe.

**Methods:**

To identify an RA biomarker profile, the 16S ribosomal DNA of fecal samples from RA patients, first-degree relatives (to rule out environment/background as confounding factors), and random healthy non-RA controls were sequenced. Analysis of metabolites and their association with specific taxa was performed to investigate a potential mechanistic link. The role of an RA-associated microbe was confirmed using a human epithelial cell line and a humanized mouse model of arthritis.

**Results:**

Patients with RA exhibited decreased gut microbial diversity compared with controls, which correlated with disease duration and autoantibody levels. A taxon-level analysis suggested an expansion of rare taxa, *Actinobacteria*, with a decrease in abundant taxa in patients with RA compared with controls. Prediction models based on the random forests algorithm suggested that three genera, *Collinsella*, *Eggerthella*, and *Faecalibacterium*, segregated with RA. The abundance of *Collinsella* correlated strongly with high levels of alpha-aminoadipic acid and asparagine as well as production of the proinflammatory cytokine IL-17A. A role for *Collinsella* in altering gut permeability and disease severity was confirmed in experimental arthritis.

**Conclusions:**

These observations suggest dysbiosis in RA patients resulting from the abundance of certain rare bacterial lineages. A correlation between the intestinal microbiota and metabolic signatures could determine a predictive profile for disease causation and progression.

**Electronic supplementary material:**

The online version of this article (doi:10.1186/s13073-016-0299-7) contains supplementary material, which is available to authorized users.

## Background

Rheumatoid arthritis (RA) is a systemic autoimmune disease characterized by inflammation of the synovial joints. Genome-wide association studies have shown that genetic factors contribute to RA susceptibility, with genes in the major histocompatibility complex (MHC) providing the strongest association and other genetic factors providing additional risk [1]. However, low concordance of RA in monozygotic twins indicates involvement of other factors [2]—perhaps an interaction between genetic and environmental factors—in the development of RA [3]. An infectious etiology of RA has been proposed for decades, although conclusive evidence is lacking [4].

During the past decade, our understanding of the interaction between microbes and host has evolved from a passive commensal relationship to recognition that the gut microbiota is essential for maintaining immune homeostasis [5, 6]. Recent studies suggest that the aberrant immune response in RA may be associated with dysbiosis of the gut microbiota [7–10]. Alterations of the normal gut microbiome can affect mucosal immunity with a consequent effect on extra-intestinal diseases like RA [8, 9], diabetes, and obesity [11, 12]. Differences in the abundance of certain commonly present gut commensals between RA patients and those with other rheumatologic diseases, as well as with healthy controls (HCs), suggests the gut microbiota has a possible association with RA [7, 9, 10]. A role for the gut microbiota in RA pathogenesis is further supported by the success of antibiotic treatment in some RA patients [13].

There is a growing realization that gut microbes and their products may affect the adaptive immune response. Introduction of segmented filamentous bacteria restores the presence of TH17 cells and contributes to the onset of arthritis in germ-free mice [14]. Mice carrying RA-susceptible human leukocyte antigen (HLA) genes show a loss of sex- and age-dependent changes in the gut microbiota that is associated with a proinflammatory cytokine profile in the gut compared with mice with RA-resistant genes [15]. The Human Microbiome Project and other studies have documented the diversity of the gut microbiome in healthy individuals and gut-related diseases [16–19].

Association of the gut microbiota with various diseases has been reported, though the specific components of the microbiota that affect the host response leading to disease remain unknown. In this study, we show not only an association between RA and certain genera but also that the role of microbes in the pathogenesis of RA is biologically plausible. Our observations suggest that RA is characterized by an expansion of certain intestinal microbes that are present in low abundance in non-RA healthy individuals.

## Methods

### Patients’ samples

Patients attending the Rheumatology Clinic at Mayo Clinic that fulfilled the exclusion and inclusion criteria were asked to enroll in the study. Adult patients (aged 18 years or older) who met the American College of Rheumatology (ACR) 2010 classification for RA were recruited. The characteristics of the study population are given in Table 1.

**Table 1:** Characteristics of the study population

	RA (*n* = 40)	Control (*n* = 32)
Age, years, mean (median)	55.7 (54)	53.0 (52)
Female	70 %	81 %
BMI, mean (median), IQR	30.4 (30.1), 23.1–33.1	30.9 (30.6), 23.9–32.4
HLA-DR4	60 %	NA
Disease activity parameters		
Disease duration, months, mean (median)	81.6 (47.5)	
DAS28, mean (median), IQR	3.2 (2.6), 0–4.3	
HAQ, mean (median), IQR	0.6 (0.5), 0.1–1.1	
ESR, mm/h, mean (median), IQR	18.5 (11), 5–23	
CRP, mg/l, mean (median), IQR	12.7 (3.6), 3–8.6	
Patient VAS pain, mm, mean (median), IQR	37.3 (28.0), 12.8–49.5	
TJC-28, mean (median), IQR	4.2 (1), 0–6	
SJC-28, mean (median), IQR	4.3 (2), 0–8	
Autoantibody status		
RF-positive	100 %	
ACPA-positive	83 %	
RF titer, kU/l, mean (median), IQR	113 (63), 15–164	
ACPA titer, kAU/l, mean (median), IQR	110 (99), 16–250	
Medication use		
Hydroxycholorquine	25.0 %	
Methotrexate	61.2 %	
Prednisone	48.9 %	
Biological agent	34.0 %	

*ACPA* anti-citrullinated protein antibody, *BMI* body mass index, *CRP* C-reactive protein, *DAS* Disease Activity Score, *ESR* erythrocyte sedimentation rate, *HAQ* Health Assessment Questionnaire, *IQR* interquartile range, *RF* rheumatoid factor, *SJC* swollen joint count, *TJC* tender joint count, *VAS* Visual Analog Scale

At the time of enrollment, any household first-degree relatives (FDRs; *n* = 15) who consented and did not have any symptoms of inflammatory arthritis or other autoimmune diseases were also enrolled. Other controls (*n* = 17) included sex- and age-matched healthy individuals with no known history of autoimmune diseases. For convenience, FDRs + HCs are labeled as controls in the figures. Any patient or control on antibiotics, consuming probiotics, or having a known history of inflammatory bowel disease or other autoimmune diseases like diabetes and multiple sclerosis were excluded. All human studies were approved by the Institutional Review Board of Mayo Clinic. Written informed consent was received from all participants prior to inclusion in the study.

### Sample collection, 16S sequencing, metabolomics, and bioinformatics processing

Fecal samples were frozen within 24 h of their receipt. Microbial DNA was extracted from fecal samples using the MoBio PowerSoil Kit with a bead-beating step. A polymerase chain reaction (PCR) was performed using 50 ng cDNA and 0.3 μM V3-V5 barcoded primers targeting 357 F and 926R with Kapa HiFi Hotstart Ready Mix (Kapa Biosystems). Samples were pooled to equal concentrations, then sequenced on one lane of MiSeq at the Mayo Genomics Facility using the MiSeq Reagent Kit v2 (500 cycles; Illumina Inc.), generating 20 M 2x250 reads. Pre-processed sequence files were then processed by IM-TORNADO [20].

Plasma samples were used for determining metabolites by mass spectrometry coupled with liquid chromatography in Mayo Metabolomics Core facility. These data were only available for patients with RA and FDRs. Methods for analysis of microbiome and metabolomics data are detailed in Additional file 1: Statistical analyses.

### Staining for tight junction proteins

The human intestinal epithelial cell line CACO-2 (ATCC) was grown in vitro as per recommendations. Expression of the tight junction protein ZO-1 was measured by immunofluorescence using a purified anti-ZO-1 antibody (Life Technologies) as the primary antibody and fluorescein isothiocyanate (FITC)-conjugated anti-rabbit IgG (Jackson ImmunoResearch Laboratories) as the secondary antibody. Expression of ZO-1 was observed using confocal microscopy (Leica DM2500, LAS-AF) and the mean florescence intensity of ZO-1 expression was calculated using image J software.

### Collagen-induced arthritis and treatment with *Collinsella*

Animal care and experiments were conducted in accordance with the institutional guidelines and after approval from the institutional animal care and use committee. The HLA-DQ8.AEo mice used in this study have been characterized and the collagen-induced arthritis model in the HLA-DQ8 transgenic mice has been described previously [21, 22]. Arthritis was induced in DQ8 mice (*n* = 18) and, 2 weeks later, mice (*n* = 10) were treated with *Collinsella* (10^9^ bacteria suspended in 100 μl tryptic soy broth (TSB), ATCC25986 strain VPI 1003, cultured as per instructions) or with media every alternate day for 4 weeks during which time the onset and progression of arthritis was monitored. The arthritic severity of the mice was evaluated with a grading system of 0–3 for each paw as described previously [21]. The mean arthritic score was determined using arthritic animals only.

To evaluate the T-cell response to *Collinsella*-primed dendritic cells (DCs), 10 days post-immunization, splenic CD4 T cells sorted from CII-primed DQ8 mice (200 μg of CII emulsified 1:1 in complete Freund's adjuvant (CFA) were cultured in vitro in the presence or absence of CII (50 μg/ml) and DCs (pre-cultured with bacteria or supernatant of the bacterial culture). T-cell proliferation was measured by routine ^3^H-thymidine incorporation [23]. All experiments were done two to three times for reproducibility.

### Intestinal permeability

As gut permeability may be diet-dependent, all transgenic mice were kept on a standard diet. Changes in intestinal permeability were determined using 4-KDa FITC-labeled dextran. Mice were deprived of food for 3 h, then gavaged with FITC-labeled dextran (0.6 mg/g body weight). Mice were bled and serum collected 3 h later. FITC-dextran content of the sera was determined by using a microplate reader with an excitation of 490 nm and emission detection at 525 nm as reported previously [15].

### rtPCR for cytokine and chemokine expression

RNA was extracted from CACO-2 cells using RNeasy columns (Qiagen) and cDNA was prepared using the SuperScript III First Strand Synthesis System (Invitrogen). Qiagen PAHS-073A RT2 Profiler PCR Array Human Th17 Response plates were used as per the manufacturer’s instructions. The data were analyzed as per the online resources of the manufacturer from their Data Analysis Center.

### Colonization of *Collinsella*

Fecal samples collected before and at various time points (3, 6, 24, and 48 h) after gavaging mice with *Collinsella* were used to determine colonization. DNA from fecal pellets was extracted and amplified using a commercial kit (Kapa Biosystems) and PCR was done using specific primers:

- AERO-F (5′-CTTTCAGCAGGGAAGAGTCAA-3′)
- AERO-R (5′-AGCCATGCACCACCTGTATGG-3′)

#### Statistical analysis

All of the statistical analyses were performed in R-3.0.2 (R Development Core Teams). Details are given in Additional file 1: Statistical analyses.

## Results

### Disease duration and seropositivity are associated with decreased microbial diversity

**Fig. 1:**
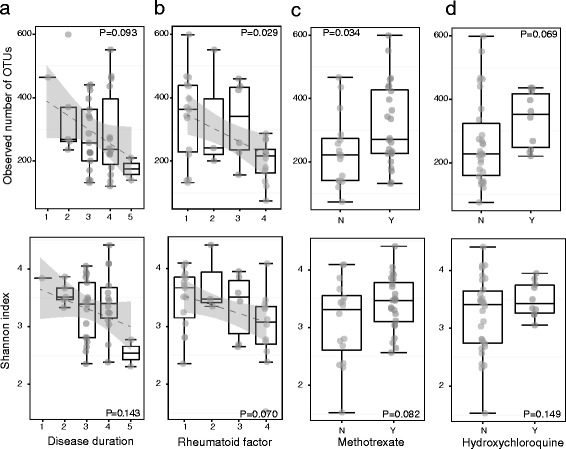
Disease duration and presence of autoantibodies correlate with α-diversity in rheumatoid arthritis patients. Two α-diversity measures, observed OTU number and Shannon diversity index, were calculated based on the rarefied counts. **a**, **b** Duration of arthritis onset (**a**) and levels of rheumatoid factor autoantibodies (**b**) in rheumatoid arthritis patients correlate with decreased α-diversity. The *dashed line* shows the fitted linear regression line with the *gray area* indicating the 95 % confidence band. Disease duration, 1 = <6 months, 2 = 6 months–1 year, 3 = 1–2 years, 4 = 2–5 years, 5= >5 years. Rheumatoid factor, 1 = <25, 2 = 25–50, 3 = 50–100, 4 = >100. **c**, **d** Treatment with methotrexate (**c**) and hydroxychloroquine (**d**) correlate with increased α-diversity. *N* not treated with specific drug, *Y* treated. The three *horizontal lines* of each *box* represent the first, second (median), and third quartile, respectively, with the *whisker* extending to 1.5 inter-quartile range. *n* = 40

The study included 40 patients with RA and 32 non-RA subjects (15 first-degree relatives (FDRs) of the probands and 17 random healthy controls (HCs). High quality 16S rDNA V3-V5 sequences obtained from fecal samples were processed by IM TORNADO (median 122,028 reads per sample, range 21,045 to 894,587) [20]. A total of 2188 operational taxonomic units (OTUs), after removing singletons, were clustered at 97 % sequence similarity (median 54 reads per OTU, range 2 to 686,387) and assigned taxonomic lineages by comparison with the greengenes 16S rDNA database (version 13.5). The OTUs were classified into 13 phyla, 26 classes, 40 orders, 76 families, and 157 genera. We first investigated the association of microbiota α- and β-diversity with the clinical variables within the RA subjects. α-Diversity determines the species richness and evenness within the microbiota while β-diversity determines the shared diversity between microbiota in terms of various ecological distances (Additional file 1: Statistical analyses). In support of previous findings [24], our data demonstrate a decreased species richness of the gut microbiota with increased body mass index (BMI; *P* = 0.025; Additional file 1: Figure S1), although no significant association with overall diversity was detected as measured by Shannon index (*P* = 0.34). Association between increased rheumatoid factor levels and disease duration with α-diversity showed decreased species richness for both clinical factors (*P* < 0.05 and *P* < 0.1, respectively) and decreased overall diversity for rheumatoid factor (*P* < 0.1; Fig. 1; Additional file 2: Table S1). Patients using methotrexate (MTX) and hydroxychloroquine exhibited an increase in species richness and diversity, indicating potential restoration of normal microbiota with treatment (*P* < 0.1; Fig. 1). Interestingly, MTX has shown interaction effects with prednisone and is associated with increased species diversity only in patients that receive prednisone (*P* < 0.05, Shannon index; Additional file 1: Figure S2). There was no association between α-diversity and HLA-DR4, radiographic erosions, or Health Assessment Questionnaire score (Additional file 2: Table S1). Disease duration, rheumatoid factor levels, C-reactive protein levels, and treatment with MTX and hydroxychloroquine were associated with β-diversity, indicating that these factors affected the gut microbiota structure.

### The gut microbiotas of patients with RA differ from those of FDRs and HCs

To determine if RA patients have a dysbiotic gut microbiota, we compared the 16S sequences of RA patients with controls (15 FDRs with no autoimmune disease and 17 randomly enrolled HCs; Table 1; Additional file 1: Figure S3). UniFrac analysis demonstrated that the microbiota of the FDRs was not significantly different from that of HCs (*P* > 0.1), and the average distance between FDRs and HCs was smaller than that between FDRs and RA patients (Additional file 1: Figure S4), indicating that disease status had larger effects than genetic and environmental factors. No significant correlation of the microbiota between FDRs and RA patients (*P* = 0.40) was observed. We thus pooled FDRs and HCs as a single control group to improve statistical power and identify consistent change.

**Fig. 2:**
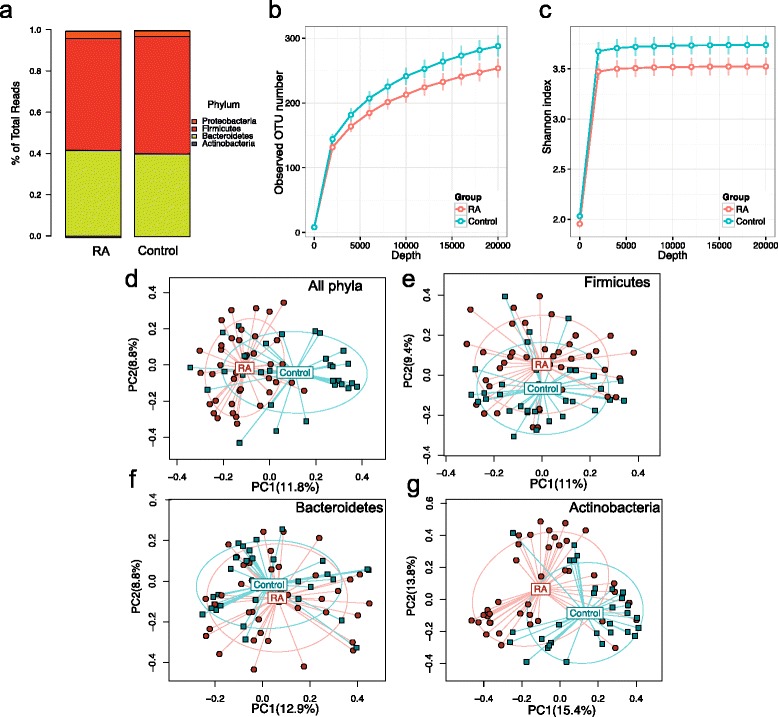
The gut microbiota of RA patients differs from that of controls. **a** Percentage of 16S reads of major phyla of the gut microbiota of RA patients and controls. **b**, **c** Rarefaction curves comparing the species richness (observed OTU numbers) (**b**) and the overall diversity (Shannon diversity index) (**c**) of RA patients and controls. The microbiota of RA patients exhibits significantly lower diversity. **d**–**g** Principal coordinate analysis plot based on the Bray–Curtis distance matrix constructed using OTUs from all phyla (**d**), *Firmicutes* (**e**), *Bacteroidetes* (**f**), and *Actinobacteria* (**g**). The percentage of variability explained by the corresponding coordinate is indicated on the *axis*. Each *point* represents a sample, *red symbols* indicate RA patients, and *blue symbols* indicate controls. The *blue lines* indicate vectors representing the relationships between the OTUs and each sample category. The *ellipses* serve a visual guide to group differences. *PC* principal component

The phylum-level profiles for RA patients and controls were rather similar, with the exception of increased number of reads from the phylum *Actinobacteria* in the RA group (0.45 vs 0.04 %, respectively; Fig. 2a). Patients with RA exhibited a significant decrease in gut microbial diversity compared with controls as observed by a decrease in OTUs and a smaller Shannon diversity index (*P* < 0.05; Fig. 2b, c). Permutational Multivariate Analysis of Variance (PERMANOVA) based on Bray–Curtis distance showed that the structure of the microbiota of RA patients differed significantly from that of controls (*P* < 0.001, 1000 permutations; Fig. 2d). Principal coordinate analysis based on phylum-specific Bray–Curtis distances revealed that microbiota from patients and controls differed much more in the low-abundant phylum *Actinobacteria* than in the two dominant phyla, *Firmicutes* and *Bacteroidetes* (Fig. 2e–g). PERMANOVA also demonstrated significant differences between the RA gut microbiota and the non-RA controls (Additional file 1: Figure S5). However, this difference was significant only in unweighted UniFrac (*P* = 0.02 and 0.4 for unweighted and weighted UniFrac, respectively), suggesting that the major microbiota difference was in the presence and abundance of rare and less abundant taxa [25].

### Expansion of rare microbial lineages characterizes the RA gut microbiota

**Fig. 3:**
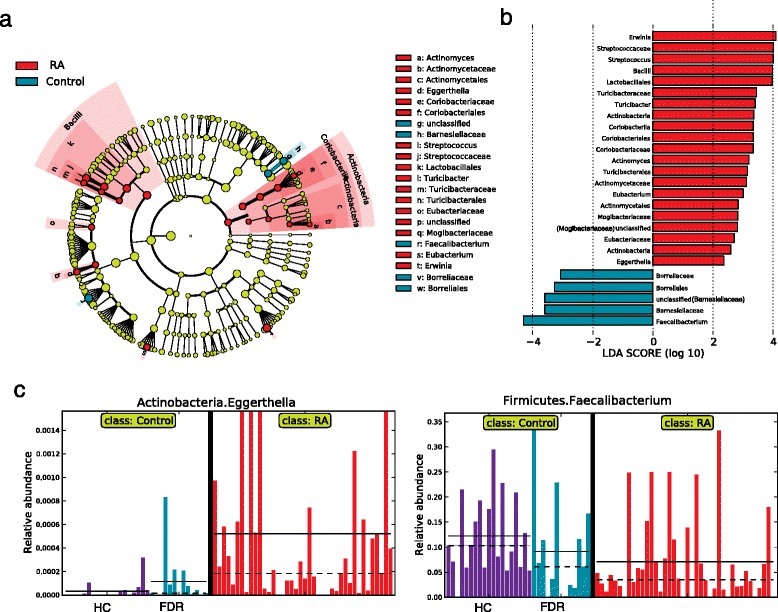
Patients with RA are characterized by expansion of rare microbial lineages. **a**, **b** LefSe analysis was performed to identify differentially abundant taxa, which are highlighted on the phylogenetic tree in cladogram format (**a**) and for which the LDA scores are shown (**b**). *Red* and *green colors* indicate an increase or decrease in taxa, respectively, in the RA patients compared with controls. Among the identified taxa, the association of the genus *Eggerthella* was the most significant and remained significant after Bonferroni correction for multiple testing. The genus *Faecalibacterium* had the largest LDA score. **c** Representation of the relative abundances of *Eggerthella* and *Faecalibacterium* in RA patients, first-degree relatives (*FDR*) and healthy controls (*HC*). Each *bar* represents the abundance of a given sample. *Solid* and *dashed lines* indicate mean and median, respectively

Using LFfSe analysis [26], we observed 26 differentially abundant taxa at different taxonomic levels. The identified taxa were highlighted on a cladogram to reveal the phylogenetic clustering pattern along with their logarithm linear discriminant analysis (LDA) scores, which measure the magnitude of differentiation between patients and controls (Fig. 3a, b; Additional file 2: Table S2). Consistent with our previous analysis, the abundance of the phylum *Actinobacteria*, along with its two genera, *Eggerthella* and *Actinomyces*, was increased in patients compared with controls. *Eubacterium* of the family *Clostridiales* and the taxonomic clade *Bacilli* from the phylum *Firmicutes* also showed clustered differences, with its two genera, *Turicibacter* and *Streptococcus*, expanded in RA patients. The genus *Eggerthella* demonstrated the most significant association with RA, which remained significant even after conservative Bonferroni correction for multiple testing was applied (*P* = 1.4e-5; Additional file 2: Table S2). In contrast to the expansion of many low-abundance microbial lineages in the RA patients, only a few taxa exhibited a decrease in abundance; the common genus *Faecalibacterium* had the largest LDA score. The relative abundance of *Eggerthella* and *Faecalibacterium* were consistently different between RA patients and controls (HCs and FDRs), confirming the above observations (Fig. 3). To address the concern of multiple testing, we applied a false discovery control to the tested associations. At a false discovery rate of 0.15, nine of the associations still remained significant (Additional file 2: Table S3; Additional file 1: Figure S6). The potential association of *Prevotella copri* as previously reported with new-onset untreated RA and DR4 [9] was not observed in this cohort of RA patients (Additional file 1: Figure S7).

We applied PICRUSt [27] to infer the functional content of the microbiota. Among 26 KEGG (Kyoto Encyclopedia of Genes and Genomes) pathways tested (Additional file 2: Table S4), the amino acid metabolism pathway exhibited differences between RA patients and controls; specifically, a decrease in OTUs with amino acid metabolism capabilities was measured in RA patients compared with controls (unadjusted *P* = 0.03; Additional file 1: Figure S8).

### Predictive modeling of the gut microbial profile for RA

**Fig. 4:**
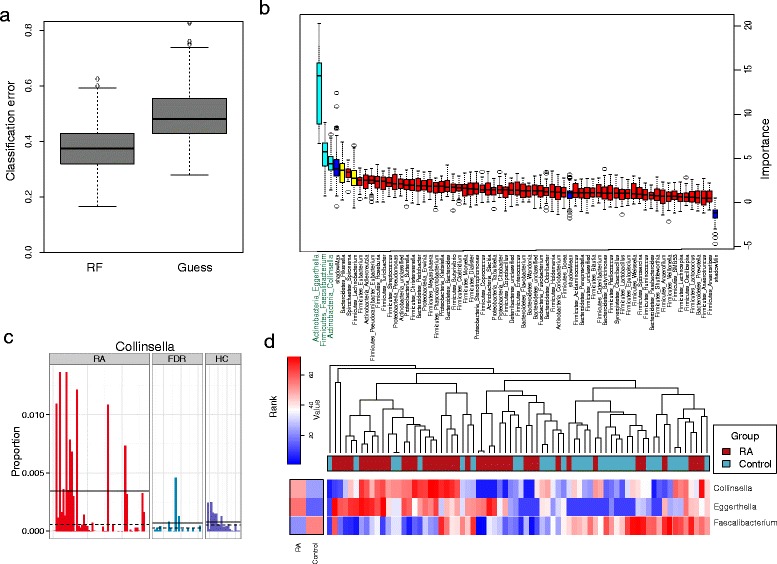
Prediction model of the gut microbiota for RA status based on the genus-level relative abundances using random forests. **a** Comparison of the classification error of the random forests-trained model with guessing, which always predicts the class label based on the majority class in the training data set. The *boxplots* are based on the results from 200 bootstrap samples. Random forests achieved a significantly lower classification error. **b** Predictive power of individual genera as assessed by the Boruta feature selection algorithm. *Blue boxplots* correspond to minimal, average, and maximum importance Z scores of shadow genera, which are shuffled versions of real genera introduced to the random forests classifier and provide a benchmark to detect truly predictive genera. *Red*, *yellow* and *cyan colors* show the rejected, tentative, and confirmed genera, respectively, by Boruta selection. Three genera, *Eggerthella*, *Faecalibacterium*, and *Collinsella*, were confirmed by Boruta selection. The genus *Collinsella* was not identified by univariate tests. **c** Many RA samples exhibit a large increase in the abundance of *Collinsella*. Solid and dashed lines indicate mean and median values respectively. ** d** Heat map based on the abundance ranks of the three Boruta-confirmed genera. *Red* and *blue* indicate high and low abundance, respectively. Hierarchical clustering (Euclidean distance, complete linkage) shows that RA samples tend to cluster together

We next used the machine learning random forests algorithm to construct a prediction model [28] (Additional file 1: Statistical analyses). Due to its non-parametric assumptions, random forests is able to detect both linear and nonlinear effects and potential taxon–taxon interactions, thereby identifying taxa that discriminate RA subjects from control subjects. To assess the prediction accuracy, we used bootstrap sampling to train the classifier and predict the class label on different subsets of samples. We achieved a mean classification error of 0.38, compared with 0.47 based on guessing (Fig. 4a; *P* < 2.2E^-16^). The importance of genera assessed by random forests generally agreed with the single-taxon-based test (Additional file 2: Tables S3 and S5). We next applied Boruta feature selection, which is a feature selection method built around random forests and selects features that have significantly more discriminatory power than random permuted features [29]. The Boruta method selected three confirmed genera: *Eggerthella*, *Faecalibacterium*, and *Collinsella* (Fig. 4b). While single-taxon tests confirmed the differences in the abundance of *Faecalibacterium* and *Eggerthella* in RA patients compared with controls, the random forests also identified differences in the genus *Collinsella* from phylum *Actinobacteria*, suggesting the potential power gain of random forests in modeling nonlinear and interactive effects. The abundance of *Collinsella* was increased in RA patients compared with controls (Fig. 4c). Hierarchical clustering based on the abundance profile of the three confirmed genera demonstrated that the samples from patients generally clustered together (Fig. 4d). Random forests analysis of species-level OTUs resulted in an even lower classification error of 0.30 (Additional file 1: Figure S9a). Many OTUs from the genera *Eggerthella*, *Collinsella*, and *Faecalibacterium* were represented in the OTUs selected by the Boruta algorithm (Additional file 1: Figure S9b, c).

### The metabolome is associated with the intestinal microbiota in patients with RA

**Fig. 5:**
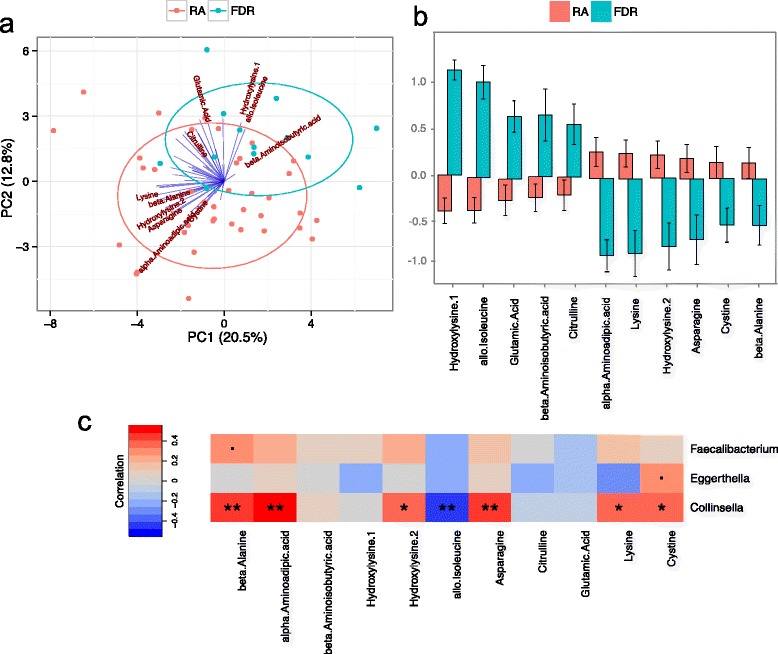
Association of plasma metabolite levels with RA disease status and gut microbiota. **a** A principal component analysis revealed that the overall metabolite profile differs between the RA patients and their first-degree relatives (*FDR*). Each point represents a sample colored by their group membership. The percentage of variance explained by corresponding principal components (*PC*) are shown on the *axes*. The direction and length of the *blue lines* indicates the contribution of the corresponding metabolites to the PCs. The *ellipses* represent a visual guide to group differences. **b** Differentially abundant metabolites between RA patients and FDRs (adjusted *P* < 0.05). The *y-axis* represents the standardized metabolite level. The *error bars* indicate the standard error of the mean. **c** A heat map shows the correlation between the abundances of the three genera *Collinsella*, *Eggerthella*, and *Faecalibacterium* and the differentially abundant metabolites. *Colors* indicate the Spearman rank correlation (**unadjusted *P* < 0.01, *P < 0.05, small black squares indicate *P* < 0.1). The differentially abundant metabolites show strong correlation with the abundance of *Collinsella*

The blood levels of 44 metabolites were measured in both RA patients and their FDRs (*n* = 53; Additional file 2: Table S6). The overall metabolomic profile differed significantly between them (*P* < 0.001; Fig. 5a). No significant correlation of the metabolome between RA patients and their FDRs (*P* = 0.75) was observed, indicating that genetic and environmental factors explain only a small percentage of the observed metabolome variability. The levels of 11 metabolites exhibited significant differences between RA patients and FDRs (adjusted *P* < 0.05; Fig. 5b), of which six were present at higher levels in the RA patients. The metabolome correlated significantly with the microbiota of RA patients (*P* = 0.03). An association of the 11 differentially abundant metabolites with the three Boruta-selected genera showed an abundance of *Collinsella* correlated with high levels of three metabolites (beta-alanine, alpha-aminoadipic acid, and asparagine), while exhibiting an inverse relationship with allo-isoleucine (*P* < 0.01; Fig. 5c; Additional file 1: Figure S10).

### *Collinsella* enhances disease severity in humanized mice

**Fig. 6:**
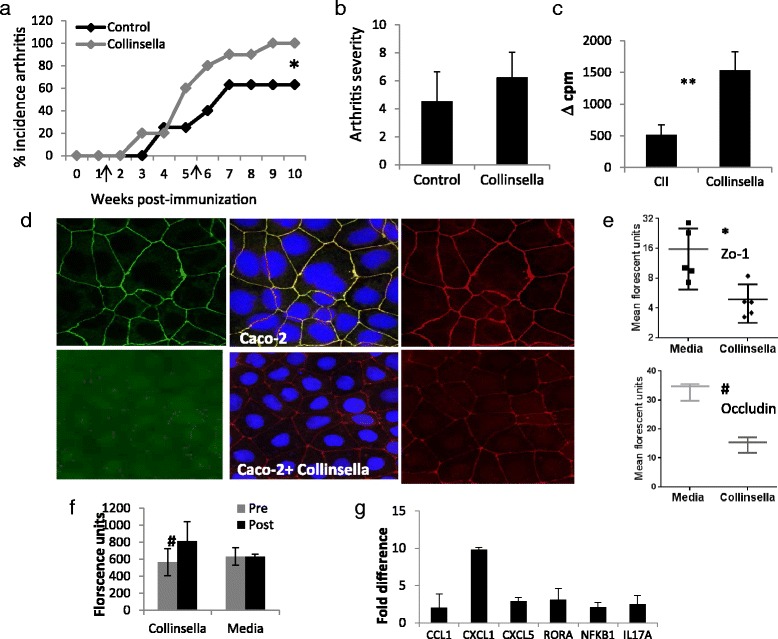
*Collinsella aerofaciens* enhances arthritis severity*.* Two weeks post-immunization (marked with *arrow*) a subset of mice were treated with *C. aerofaciens* every alternate day for 4 weeks (marked with *arrows*), *n* = 10. Mice not treated with *C. aerofaciens* (*n* = 8) were used as a control. Mice were followed for **a** incidence and onset of arthritis (*P = 0.068) and **b** disease severity. *Collinsella* enhances T-cell proliferation. **c** T-cell proliferation was measured by culturing sorted (by fluorescence-activated cell sorting) CD4 cells from the spleens of CII-primed mice cultured with dendritic cells that were pre-cultured with *Collinsella* for 4 h. **P = 0.02 (*n* = 3 mice/group). *Collinsella* reduces the expression of the tight junction protein ZO-1 and Occludin. **d** CACO-2 cells cultured with or without *Collinsella* stained with ZO-1 and Occludin showed differences in the expression of tight junction proteins. **e** Quantification of the mean fluorescence intensity of ZO-1 and Occludin expression in CACO-2 cells cultured alone or in the presence of *Collinsella*, ^#^ *P* < 0.05 and *P < 0.01. **f** Increased gut permeability was observed in DQ8 mice when *Collinsella* was administered. Sera of mice were tested for FITC-Dextran before and after treating mice with *Collinsella* for 3 weeks (*P = 0.03; *n* = 10 mice/group). **g** Fold difference in the expression of Th17 regulatory cytokine/chemokine transcripts in CACO-2 cells cultured with C*. aerofaciens* compared with CACO-2 cells cultured with bacterial growth media. Error bars represent standard error of the mean values. Experiments were repeated for reproducibility

The pathogenicity of *Collinsella* was confirmed in a humanized mouse model. Collagen-induced arthritis-susceptible HLA-DQ8 mice were treated with *Collinsella aerofaciens* (*n* = 10) and compared with untreated mice (*n* = 8). Mice given *C. aerofaciens* developed arthritis with increased incidence and severity compared with non-treated mice (100 % incidence in treated vs 62.5 % in untreated, *P* = 0.068), although disease severity did not differ significantly (Fig. 6a, b). However, no colonization by the microbe was observed (Additional file 1: Figure S11). To determine the influence of *Collinsella* on the immune response, we tested the antigen-specific recall response by culturing splenic CD4 cells of type II collagen (CII)-primed DQ8 mice with untreated dendritic cells (DCs) or those that were pre-cultured in vitro with *Collinsella* (*n* = 3/group). A significantly robust CD4 T-cell response to CII was detected in the presence of DCs that were pre-cultured with the bacteria compared with the response with untreated DCs (*P* = 0.02; Fig. 6c).

### *Collinsella* increases gut permeability by reducing the expression of tight junction protein in epithelial cells and induces expression of IL-17 network cytokines

For applicability to RA, a human intestinal epithelial cell line, CACO-2, was cultured in the presence or absence of *Collinsella* (Fig. 6d). *Escherichia coli* was used as a control. Our observations suggested a significant decrease in the expression of the tight junction protein ZO-1 in the presence of *Collinsella* as determined by staining and mean florescence intensity (Fig. 6d, e) while *E. coli* did not show a significant difference compared with the media control (Additional file 1: Figure S12). To determine if *Collinsella* lowers gut permeability, we compared gut permeability before and after administering media (*n* = 10) or *Collinsella* (*n* = 10) for 3 weeks. *Collinsella* administration led to a significant increase in gut permeability compared with pre-treatment (*P* < 0.05; Fig. 6f). No change in gut permeability was observed with *E. coli* (Additional file 1: Figure S12). We also determined if *Collinsella* induced mRNA expression of Th17 regulatory network cytokines in CACO-2 cells (*n* = 3; Fig. 6g). Compared with controls, culturing with *Collinsella* led to more than a twofold increase in the expression of interleukin (IL)-17A as well as RORα and chemokines CXCL1 and CXCL5, which are known to regulate production of IL-17 [30, 31]. Also, an increase in NFkB1 expression suggested activation of inflammatory pathways. These data suggest that an expansion of *Collinsella* may cause an increase in pro-inflammatory conditions with a loss of gut epithelial integrity.

## Discussion

Accumulating evidence suggests that RA is a multifactorial disease dependent on an interaction between genetic and environmental factors [32, 33]. The HLA-DRB1 “shared epitope” provides the highest genetic risk factor for RA patients [1]. Environmental factors affecting mucosal surfaces (smoking and infections) have the most influence on RA pathogenesis [10].

There is growing speculation about the role of the gut in systemic autoimmune diseases. Humanized mice expressing an RA-susceptible HLA gene exhibited a dysbiotic fecal microbiome compared with mice carrying an RA-resistant gene [15]. A recent study detected an abundance of the bacteria *P. copri* in fecal samples of patients with new-onset RA, suggesting a role for the gut microbiome in RA [9]. Interestingly, an inverse relationship between the presence of *P. copri* and the presence of a shared epitope was observed, suggesting that this bacterium may contribute to pathogenesis in a subset of patients. We did not observe a difference in either the abundance of *Prevotellaceae* or *P. copri* or their associated OTUs between RA patients and controls (Additional file 1: Figure S7). In contrast to the previous study, however, all the patients in the present study were currently on a treatment regimen. Association of disease severity measures with the gut microbiota of patients showed rheumatoid factor levels and disease duration to be associated with the decreased species richness after adjusting for various drugs used for treatment. Together, the present and previous data suggest that cohorts with different patient characteristics, including disease stage (i.e., early versus established), activity, and geographical locations, may show different microbial associations. Microbial metabolites may provide a window to the functioning of the microbiota and assume universal importance.

Autoreactive T-cell responses and auto-antibody production leading up to the onset of RA occur much earlier than the clinical presentation of RA [34]. Since random HCs possess different genetic factors to the RA patients, we enrolled FDRs as additional controls so the major effects observed would be driven by environmental factors or due directly to the disease process. This would help in elucidating the expansion or contraction of specific bacterial clades in RA patients. Interestingly, FDRs did not differ significantly from HCs in their fecal microbiota, suggesting that differences in certain taxa, such as those observed in the current study, may be dependent on disease state or factors other than genetics, although genetic factors may contribute to an altered state of the immune response. However, the sample size for FDRs was small, which may have limited the power of analysis.

Our data suggest that the differential microbial community structure between RA patients and controls was driven by differences in taxa, mainly the presence and abundance of rare and less abundant lineages. The predictive model suggested that microbes belonging to the phylum *Actinobacteria* play a significant role in RA pathogenesis as both *Collinsella* and *Eggerthella* were observed to predict the RA status. The abundances of *Eggerthella* and *Collinsella* were not significantly associated with the three commonly used drugs, methotrexate (MTX), prednisone, and hydroxychloroquine. These observations confirm a recent study that showed that dysbiosis in the gut microbiome in RA patients is restored partially after treatment with MTX [8]. The abundance of *Faecalibacterium* showed a significant positive association with the use of hydroxychloroquine (*P* < 0.05), which does not explain the reduced *Faecalibacterium* in RA. Overall, these observations suggest that treatments are probably not the confounding factor for the identified associations. An important role for *Collinsella* was confirmed both in vivo using a humanized mouse model of arthritis and in vitro using human intestinal epithelial cells. A recent study showed shared sequences between *Collinsella* and DRB1*0401, suggesting that *Collinsella* might contribute to RA via molecular mimicry [8], further supporting the current observations that HLA class II molecules can present self-HLA-derived peptides [35] and mimicry with a pathogen can result in enhanced stimulation and inflammation in certain conditions.

One mechanism by which *Collinsella* contributes to disease pathogenesis is by increasing gut permeability as observed by the lower expression of tight junction proteins. Additionally, *Collinsella* influences the epithelial production of IL-17A and the chemokines CXCL1 and CXCL5, which may result in recruitment of neutrophils and activation of NFkB, which has been observed to be involved in the pathologic effects of a gut pathobiont [36]. Recently, a multifactorial role of neutrophils has been suggested in RA [37]. CXCL5 production by epithelial cells in response to *Bacteroides fragilis* is associated with an inflammatory response [38]. Both CXCL1 and CXCL5 are increased in arthritis [39]. IL-17A, a major cytokine involved in arthritis, upregulates CXCL1, which is known to cause increased cell migration, angiogenesis, and activation of the STAT-3 pathway [40]. Induction of Th17 cytokines systemically by *Collinsella* would be informative about its role in arthritis but was not investigated in this study. Our data suggest that *Collinsella* contributes to hyper-permeability of the gut by reducing the expression of the tight junction protein ZO-1 directly, as well as by producing specific metabolites. In support of this, the abundance of *Collinsella* correlated strongly with high levels of beta-alanine, alpha-aminoadipic acid, and asparagine. Alpha-aminoadipic acid is a marker for autoimmunity and age-associated changes in human collagen [41, 42], while asparagine is a non-essential amino acid involved in the tricarboxylic acid cycle and blocking apoptosis [43]. Currently, the source of asparagine is unknown in this study. Age-associated changes in collagen and blocking of apoptosis could be involved in the autoreactive response to collagen in patients, though these mechanisms need to be proved.

*Eggerthella lenta* is another organism that was detected with more abundance in RA patients, using multiple methods of analysis, but only rarely in controls. *Eggerthella* uses ornithine as substrate to generate energy, producing citrulline and carbamyol phosphate as byproducts. We did not observe any association between the presence of *Eggerthella* and citrulline levels in the sera of patients. However, it is unknown whether RA patients carry higher loads of this amino acid or citrullinated peptides in the gut. Based on the higher abundance of *Eggerthella* in patients in the present study, we predict that patients with RA may exhibit increased levels of citrulline in the gut available for citrullination, against which antibodies might be produced. Carbamyol phosphate is an enzyme that is involved in the pyrimidine pathway. This pathway is upregulated in RA patients and typically treated with pyrimidine synthesis inhibitors such as leflunomide [44]. None of the patients in our study were on leflunomide. While these data provide tantalizing clues, the roles of these metabolites and rare taxa of the gut microbiome deserve further study.

The gut microbiota of RA patients exhibited decreased diversity with increased disease duration and seropositivity. This change in diversity stemmed from an expansion of rare lineages like *Eggerthella* and from a contraction of the common beneficial genera like *Faecalibacterium. Faecalibacterium* is one of the most abundant *Firmicutes* in the human gut that produces butyrate [45]. Butyrate is required for epithelial proliferation and mucin synthesis and production, which helps maintain the integrity of the gut epithelial layer. A decreased abundance of *Faecalibacterium* with increased *Collinsella* may lead to an increase in epithelial permeability, causing microbial fragments and products to enter the sub-epithelial space and lamina propria. In the presence of these conditions, a change in abundance of any microbial clade that leads to an altered immune state may cause local inflammation in the gut as well as outside the gut. The Boruta feature selection algorithm and LEfse analysis also confirmed the significance of the differential presence of *Eggerthella*, *Collinsella*, and *Faecalibacterium* in RA patients compared with controls.

Elevated BMI has a significant impact on the gut microbiota of RA patients in this study. The distribution of BMI was not significantly different between the patients and controls, suggesting obesity is unlikely to have a major confounding effect on the differences in the gut microbiota between patients. Obesity and elevated BMI are associated with both the incidence [46, 47] and prognosis of RA [48]. Therefore, restricting the sample of patients to those with healthy weight might have inappropriately limited the generalizability of our findings, though we cannot exclude the possibility of confounding by BMI in this study. Future prospective longitudinal studies are warranted to dissect the potential interactions of obesity and gut microbiota on the pathophysiology of RA.

One can envisage that in a healthy state, dynamic microbiome structures based on sex, diet, and other factors and driven by specific bacterial groups, maintain homeostasis that modulates the immune response. In contrast, this kind of microbial axis dynamism is lost in patients. Although specific molecular mechanisms remain largely unexplored, the results of this study suggest that susceptibility to RA could be triggered by gut dysbiosis and alterations in pathways in which rare lineages are involved. However, the study needs to be confirmed with a larger patient and FDR cohort. An interesting observation was the loss of sex-biased differences in RA patients, as a healthy human microbiota is sex-dependent [49]. In the present study, there were not enough males in the HC group to perform an analysis of sufficient power to evaluate this factor. Our studies support previous data that showed a loss of sex bias in the fecal microbiota of genetically arthritis-susceptible humanized mice [15]. Further, similar to the humanized mice, expansion of certain taxa was observed in RA patients.

## Conclusions

Collectively, our data demonstrate that a dysbiotic gut microbiota in RA patients, characterized by a decrease in *Faecalibacterium* and expansion of *C. aerofaciens* and *E. lenta*, could trigger inflammatory conditions in the gut that depend on the production of chemokines and IL-17A and compromise the gut epithelium integrity. It is possible that the inflammatory conditions can be modulated by prebiotics or probiotics. The therapeutic potential of the only probiotics, lactobacilli, used as treatment for RA is inconclusive, with some studies in favor of their use while others did not show significant improvement with lactobacilli using the American College of Rheumatology (ACR) response criteria for RA [50–53]. Our data suggest specific microbial clades may be viable targets for therapeutic manipulation by diet, probiotics, prebiotics, and/or beneficial gut commensals. Determining the functions of the microbial clades that expand or contract in RA will assist in developing effective means to target them.

### Ethics approval

All human studies were approved by the Institutional Review Board of Mayo Clinic and conducted in accordance with the Helsinki Declaration. Written informed consent was received from all participants prior to inclusion in the study. Animal care and experiments were conducted in accordance with and after approval from the Institutional animal care and use committee.

### Availability of data and materials

Data can be accessed via BioProject PRJNA317370.

## Additional files

Additional file 1: Statistical analysis. **Figure S1.** Body mass index (*BMI*) was associated with decreased species richness in the gut microbiota of RA patients. Species richness was measured by the observed OTU numbers and calculated on the rarefied counts. The *dashed line* shows the fitted linear regression line with the *gray area* indicating the 95 % confidence band. The three *horizontal lines* of the *box* represent the first, second (median), and third quartiles, respectively, with the *whisker* extending to 1.5 inter-quartile range (IQR). BMI-1 ≤ 24, 2 ≤ 30, 3 ≤ 35, 4 ≤ 40 **Figure S2.** Treatment effects (methotrexate, hydroxychloroquine, or either) on microbiota α-diversity, stratified by prednisone use. *N* not treated with specific drug, *Y* treated. The three *horizontal lines* of each *box* represent the first, second (median), and third quartile, respectively, with the *whisker* extending to 1.5 inter-quartile range. *n* = 40. **Figure S3.** Heat map showing the genus-level profiles of the gut microbiota of RA, their first-degree relatives (*FDR*) and healthy controls (*HC*). The heat map colors indicate the abundance of the genera. **Figure S4.** Boxplots comparing the inter-group UniFrac distances (RA versus first degree relatives (*FDR*), RA versus controls and FDRs versus healthy controls (*HC*)). The distances between relatives and HCs are smaller than those between RA and relatives or HCs, indicating disease may have stronger effects on the gut microbiota than genetic or environmental factors. **Figure S5.** Principal coordinate plots based on unweighted and weighted UniFrac distances. PERMANOVA analysis showed a significant difference between RA patients and controls on unweighted UniFrac (**a**) instead of weighted UniFrac (**b**), indicating the microbiota change in RA mostly occurs in the rare and less abundant lineages. The percentage of variability explained by the corresponding coordinate is indicated on the *axis*. Each point represents a sample with *red* and *blue color* indicating the RA and control groups, respectively. The *lines* connecting to the centroid and the *ellipses* do not represent any statistical significance but rather serve a visual guide to group differences. **Figure S6.** Comparison of the relative abundances of differentially abundant taxa between RA and controls selected based on a false discovery rate of 15 %. *Error bars* represents the standard error of the mean. The *y-axis* is on squared-root scale. **Figure S7.** The relative abundance of *Prevotella copri* does not show significant difference between RA and controls. **a** The presence of *P. copri* OTUs was similar between RA and controls. The row names of the heat map depict the *P. copri* OTUs. *Red color* indicates the presence of the OTUs. **b** The relative abundance of the *P. copri* OTUs is similar between RA patients and controls. **c** The presence and abundance of *P. copri* does not correlate with the presence or absence of HLA-DR4 in RA patients. **Figure S8.** The RA gut microbiota has decreased function in amino acid metabolism. The abundance of the KEGG pathway categories was calculated based on PICRUSt. The three *horizontal lines* of the *box* represent the first, second (median), and third quartile, respectively, with the *whisker* extending to the 1.5 inter-quartile range (IQR). **Figure S9.** The predictive power of gut microbiota profile (species) for RA status assessed by machine learning algorithm random forests. Random forests, an ensemble classifier built upon many decision trees, was used to build a prediction model based on the OTU-level relative abundances. **a** Comparison of the classification error of the random forests-trained model with guessing, which always predicts the class label based on the majority class in the training data set. The box plots are based on the results from 200 bootstrap samples. Random forests achieves significantly lower classification error. **b** Predictive power of individual OTUs assessed by the Boruta feature selection algorithm. *Deep blue box plots* correspond to the maximum importance Z score of shadow genera, which are shuffled versions of real genera introduced to the random forests classifier and provide a benchmark to detect truly predictive genera. *Yellow* and *light blue* colors show the tentative and confirmed genera by the Boruta selection. **c** Heat map based on the abundance ranks of the three Boruta-confirmed OTUs. *Red* and *blue colors* indicate high and low abundance, respectively. Hierarchical clustering (Euclidean distance, complete linkage) shows that RA samples tend to cluster together. **Figure S10.** Scatterplots showing the correlation of the abundance of differentially abundant metabolites with *Collinsella* abundance. Significance was assessed by Spearman rank correlation test. The *blue line* shows the fitted linear regression with the *gray area* indicating the 95 % confidence band. **Figure S11.** *Collinsella* does not colonize the gut. Fecal pellets collected before mice were gavaged with *C. aerofaciens* and at various time points (3, 6, 12, 24, and 48 h) after gavage were collected and PCR was done to determine the presence of the microbe. DNA from *C. aerofaciens* was used as a positive control and culture media as a negative control. After 12 h of gavage, a very faint band was observed and after 24 h, *C. aerofaciens* was not detectable in fecal pellets, suggesting the microbe did not colonize the intestine. **Figure S12.** *E. coli* does not significantly alter the gut permeability. *E coli* was used as a control microbe for gut permeability. **a** Gut permeability in DQ8 mice administered *E. coli* or media did not show significant change. Sera of mice were tested for FITC-Dextran before and after treating mice with *E. coli* for 3 weeks (*P* = not significant, *n* = 6 mice/group). **b** CACO-2 cells cultured with or without *E. coli* stained with ZO-1 showed no significant difference in the expression of tight junction protein. **c** Quantification of the mean fluorescence intensity of ZO-1 expression in CACO-2 cells cultured in the presence of *E. coli* or media (*P* = not significant). (PDF 2660 kb) Additional file 2: Table S1. Association of clinical variables with gut microbial diversity. The association of α-diversity was assessed by a linear model and the association of β-diversity was assessed by PERMANOVA. **Table S2.** Differential abundance analysis of phylum, family, and genus-level taxa. **Table S3.** Summary statistics of differentially abundant taxa at a false discovery rate of 15 %. **Table S4.** Differential abundance analysis results of major KEGG pathways. **Table S5.** Importance scores of the genera determined by the random forests algorithm. **Table S6.** Abundance of metabolites in RA and relatives. Mean and standard deviation are given. (XLSX 36 kb)

## Abbreviations

BMI: body mass index
CII: type II collagen
DC: dendritic cell
FDR: first-degree relative
FITC: fluorescein isothiocyanate
HC: healthy control
IL: interleukin
KEGG: Kyoto Encyclopedia of Genes and Genomes
LDA: linear discriminant analysis
MTX: methotrexate
OTU: operational taxonomic unit
PCR: polymerase chain reaction
PERMANOVA: Permutational Multivariate Analysis of Variance
RA: rheumatoid arthritis
