# Supplementary material for: An expansion of rare lineage intestinal microbes characterizes rheumatoid arthritis
Source: Genome Med. 2016 Apr 21;8:43. doi: 10.1186/s13073-016-0299-7 (PMC4840970; doi:10.1186/s13073-016-0299-7)
Supplement: Additional file 1: — Statistical analysis. Figure S1. Body mass index (BMI) was associated with decreased species richness in the gut microbiota of RA patients. Species richness was measured by the observed OTU numbers and calculated on the rarefied counts. The dashed line shows the fitted linear regression line with the gray area indicating the 95 % confidence band. The three horizontal lines of the box represent the first, second (median), and third quartiles, respectively, with the whisker extending to 1.5 inter-quartile range (IQR). BMI-1 ≤ 24, 2 ≤ 30, 3 ≤ 35, 4 ≤ 40 Figure S2. Treatment effects (methotrexate, hydroxychloroquine, or either) on microbiota α-diversity, stratified by prednisone use. N not treated with specific drug, Y treated. The three horizontal lines of each box represent the first, second (median), and third quartile, respectively, with the whisker extending to 1.5 inter-quartile range. n = 40. Figure S3. Heat map showing the genus-level profiles of the gut microbiota of RA, their first-degree relatives (FDR) and healthy controls (HC). The heat map colors indicate the abundance of the genera. Figure S4. Boxplots comparing the inter-group UniFrac distances (RA versus first degree relatives (FDR), RA versus controls and FDRs versus healthy controls (HC)). The distances between relatives and HCs are smaller than those between RA and relatives or HCs, indicating disease may have stronger effects on the gut microbiota than genetic or environmental factors. Figure S5. Principal coordinate plots based on unweighted and weighted UniFrac distances. PERMANOVA analysis showed a significant difference between RA patients and controls on unweighted UniFrac (a) instead of weighted UniFrac (b), indicating the microbiota change in RA mostly occurs in the rare and less abundant lineages. The percentage of variability explained by the corresponding coordinate is indicated on the axis. Each point represents a sample with red and blue color indicating the RA and control groups, respec [file 13073_2016_299_MOESM1_ESM.pdf]

Online Methods Supplement Data

Statistical analyses

α-diversity analysis: α-diversity determines the species richness and evenness within bacterial populations. Two α-diversity metrics, observed OTU number and Shannon index, were calculated based on the rarefied OTU counts to address differential sequencing depths (‘estimate richness’ function in Bioconductor package ‘PhyloSeq’)(McMurdie and Holmes, 2013). The observed OTU number depicts the species richness, while the Shannon index, which is a measure of both species richness and evenness, is an overall measure. Rarefaction curves were created by rarefying the OTU counts to different sequencing depths. A simple linear model was used to test the association between α-diversity measures and variables of interest, adjusting for other covariates when necessary.

β-diversity analysis: β-diversity determines the shared diversity between bacterial populations in terms of various ecological distances. Different distance metrics reveal distinctive views of community structure. Unweighted, weighted and Bray-Curtis distances were constructed using the OTU table and the phylogenetic tree (R package ‘GUniFrac’)(Chen et al., 2012). UniFrac distances measure the shared phylogenetic diversity between communities. Unweighted UniFrac distance captures the difference in community membership and/or rare and less abundant lineages while weighted UniFrac distance is more influenced by the change in abundant lineages. Rarefaction was performed on the OTU table before calculating the distances. Based on these distance matrices, PERMANOVA was used to test for an association between variables of interest and the overall microbiota composition adjusting for covariates when necessary (R package ‘vegan’) (McArdle and Anderson, 2001). Significance was assessed by 1,000

permutations for all distance-based methods. PCoA was performed on the distance matrix (‘cmdscale’ in R) and the first two PCs were used to generate the ordination plots.

Differential abundance analysis: A taxonomic search using the greengenes 16SrDNA database allowed us to identify the taxonomic lineages of the OTUs. A non-parametric Wilcoxon rank-sum test was used to test group difference in taxa abundances at phylum, family and genus levels. The Benjamini-Hochberg (BH) procedure-based false discovery rate control was used to correct for multiple testing (‘p.adjust’ in R). To reduce the number of tests, we confined the analysis to taxa with prevalence > 10% and maximum proportions > 0.002. An adjusted P-value (Q-value) less than 0.15 was considered to be statistically significant. Differential abundance analysis was also performed using the LEfSe software with a nominal p-value of 0.05 and a logarithmic LDA score of 2 as cutoffs as suggested by the software(Segata et al., 2011). PICRUSt was used to infer the abundance of functional categories (KEGG pathways) based on the 16S rRNA data (Langille et al., 2013) and differential abundance analysis was performed using a Wilcoxon rank-sum test.

Predictive modeling of RA status using random forests: The machine learning algorithm random forests (Breiman, 2001) was used to classify the subjects into two classes (RA and control) based on their microbiota profile using default parameters of the R implementation of the algorithm (R package ‘randomforest’). The random forests algorithm, due to its non-parametric assumptions, was able to detect both linear and nonlinear effects and potential taxon-taxon interactions, thereby identifying taxa that discriminate RA subjects from control subjects. The genus or species-level proportion data served as input data. Bootstrapping (N=200) was used to assess the classification accuracy, where the bootstrapped samples were used as a training set and the unused samples as a test set. The classification performance was compared to guess,

where the class label for the samples in the test set was predicted to be the label of the majority class in the training set, and Friedman rank-sum test was used to test the significance of the difference. Boruta variable selection was applied to select the most discriminatory taxa based on the importance values produced by random forests (Kursa and Rudnicki, 2010). The importance value of a genus was calculated based on the loss of accuracy by the random permutation of the abundance of the taxon. The Boruta method spiked-in 'shadow' taxa, which were shuffled version of real taxa, to the real abundance data. This enabled us to assess if the importance of a given taxon is significant, that is, whether it is discernible from the importance that may arise from random fluctuations (shadow taxa). Variable selection via a combination of random forests and Boruta is expected to be more powerful than univariate (marginal) tests, such as the Wilcoxon rank-sum test, in identifying important taxa that are individually weak but jointly strong predictors of the disease state.

Metabolomic data analysis: These data were only available for patients with RA and FDRs. The metabolomic data were first subjected to normal transformation to meet the normality assumption of the statistical tests. PCA was then used to reduce the dimensionality of the metabolomic data and the subjects were projected on the first two PCs to visualize their relationships. A PERMONOVA test was used to test for the overall difference of the metabolomic profiles between patients with RA and FDRs based on Euclidean distance. A two-sample Student's t-test was then used to identify differential metabolites between RA patients and their FDRs. False discovery rate control (BH procedure) was used to correct for multiple testing. The first principal component (PC1) from the PCA analysis of the metabolome, which summarizes the overall metabolome variation pattern, was tested for an overall association with the microbiota of patients using PERMANOVA on UniFrac distance. The metabolites that

differed between patients with RA and their FDRs were also tested for specific association with the microbiota using PERMANOVA within RA patients. The specific associations between these differential metabolites and taxa were investigated using the Spearman's rank correlation test.

All of the above statistical analyses were performed in R-3.0.2 (R Development Core Teams).

Mouse data analysis: The difference in the incidence of arthritis between groups was analyzed using a Fisher's exact test. Antibody levels, onset of arthritis, and mean scores for arthritic mice were compared using the non-parametric Student's t test. All other significance was calculated using a Student's t-test or a t-test with unequal variance.

References

Breiman, L. (2001). Random Forests. Machine Learning *45*, 5–32.

Chen, J., Bittinger, K., Charlson, E.S., Hoffmann, C., Lewis, J., Wu, G.D., Collman, R.G., Bushman, F.D., and Li, H. (2012). Associating microbiome composition with environmental covariates using generalized UniFrac distances. Bioinformatics *28*, 2106–2113.

Kursa, M.B., and Rudnicki, W.R. (2010). Feature selection with the Boruta package. J Stat Softw *36*, 1–13.

Langille, M.G.I., Zaneveld, J., Caporaso, J.G., McDonald, D., Knights, D., Reyes, J.A., Clemente, J.C., Burkepile, D.E., Vega Thurber, R.L., Knight, R., et al. (2013). Predictive functional profiling of microbial communities using 16S rRNA marker gene sequences. Nat. Biotechnol. *31*, 814–821.

McArdle, B.H., and Anderson, M.J. (2001). Fitting Multivariate Models to Community Data: A Comment on Distance-Based Redundancy Analysis. Ecology *82*, 290–297.

McMurdie, P.J., and Holmes, S. (2013). phyloseq: an R package for reproducible interactive analysis and graphics of microbiome census data. PLoS ONE *8*, e61217.

Segata, N., Izard, J., Waldron, L., Gevers, D., Miropolsky, L., Garrett, W.S., and Huttenhower, C. (2011). Metagenomic biomarker discovery and explanation. Genome Biol *12*, R60.

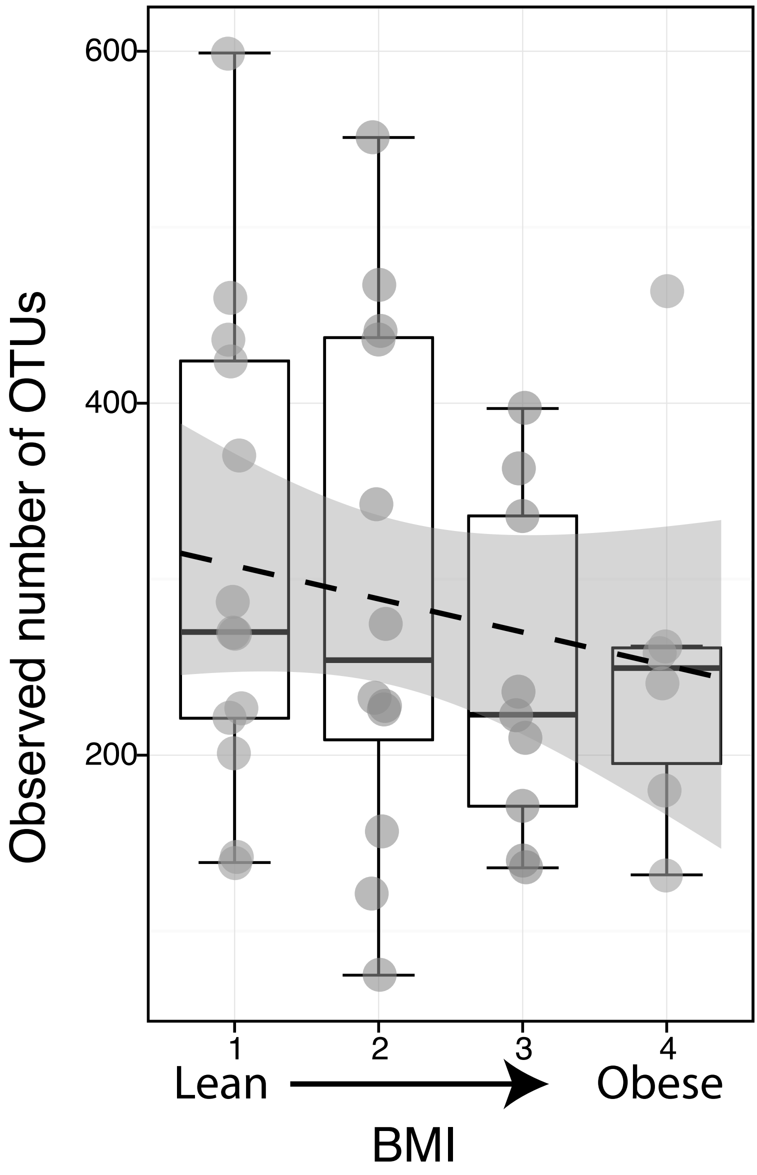

Figure S1. Body mass index (BMI) was associated with decreased species richness in the gut microbiota of RA patients. Species richness was measured by the observed OTU number, calculated on the rarefied counts. The dashed line shows the fitted linear regression line with the gray area indicating the 95% confidence band. The three horizontal lines of the box represents the first, second (median) and third quartile respectively with the whisk extending to 1.5 inter-quartile range (IQR). BMI-1≤24, 2≤30, 3≤35, 4≤40.

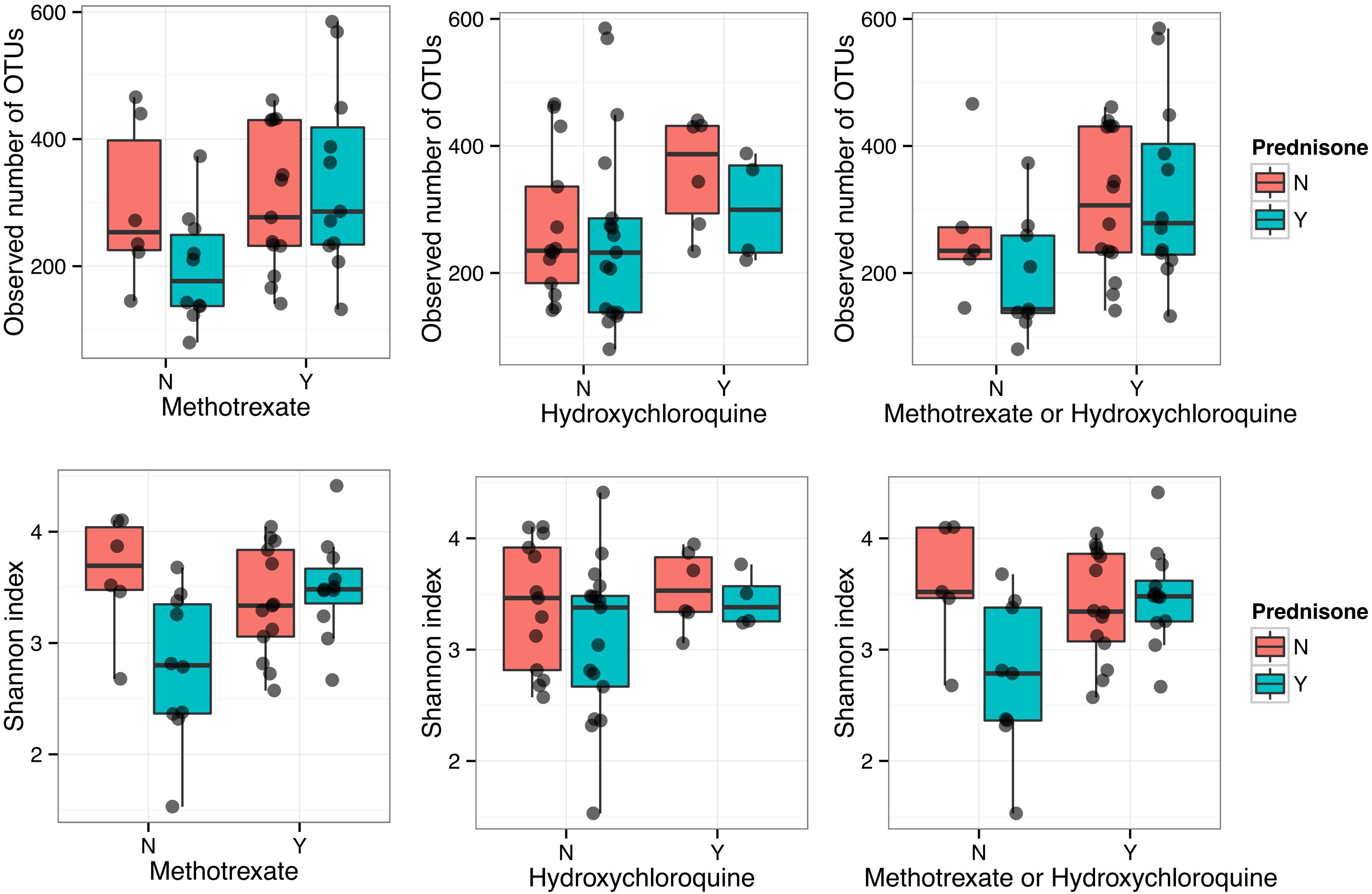

Figure S2. Treatment effects (methotrexate, hydroxychloroquine or either) on microbiota alpha-diversity, strati-fied by prednisone use. N= not treated with specific drug, Y= treated. The three horizontal lines of each box represent the first, second (median) and third quartile, respectively, with the whisk extending to 1.5 inter-quar-tile range.

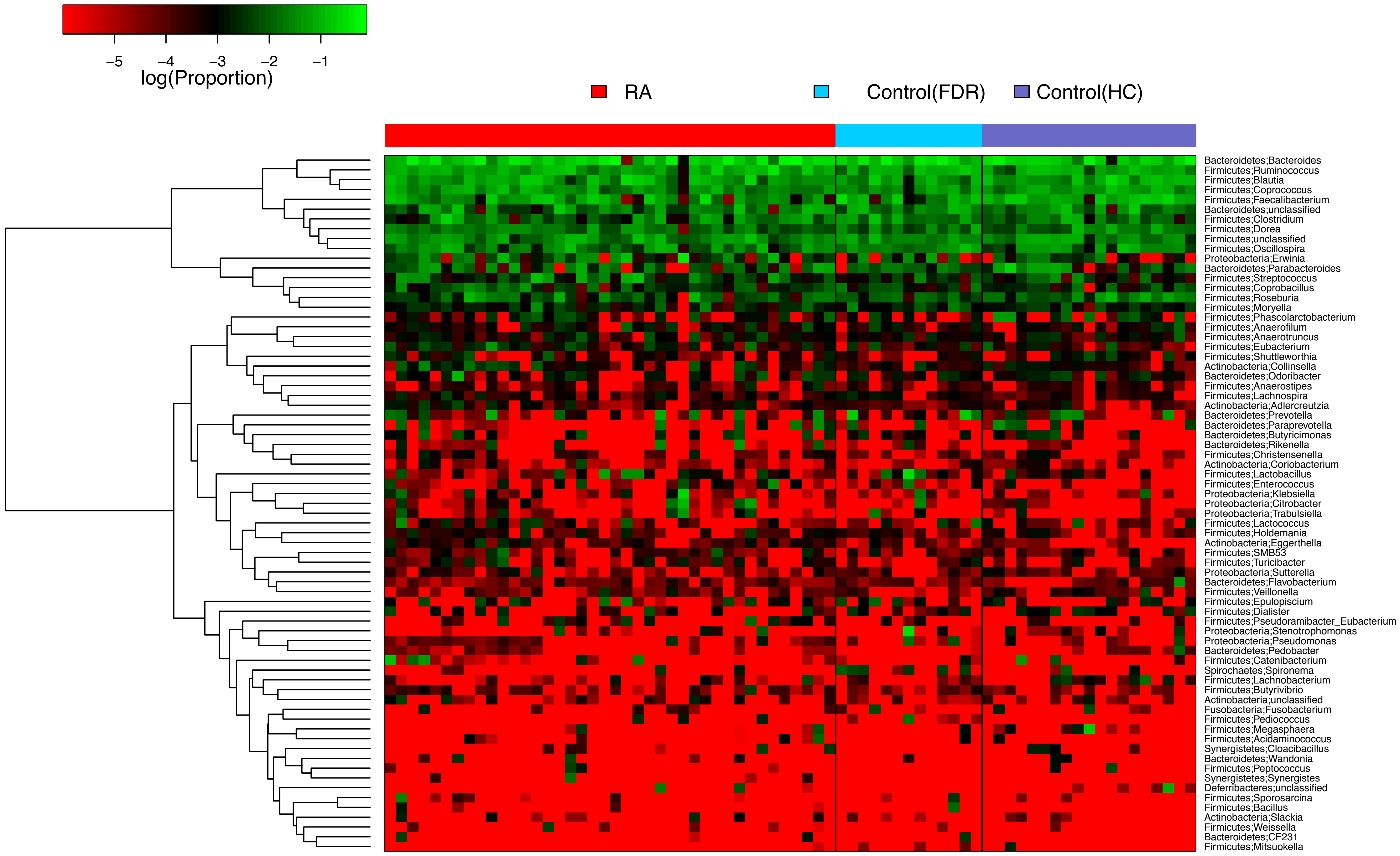

Figure S3. Heatmap showing the genus-level profiles of the gut microbiota of RA, their first-degree relatives (FDR) and healthy controls (HC). The heatmap colors indicate the abundance of the genera.

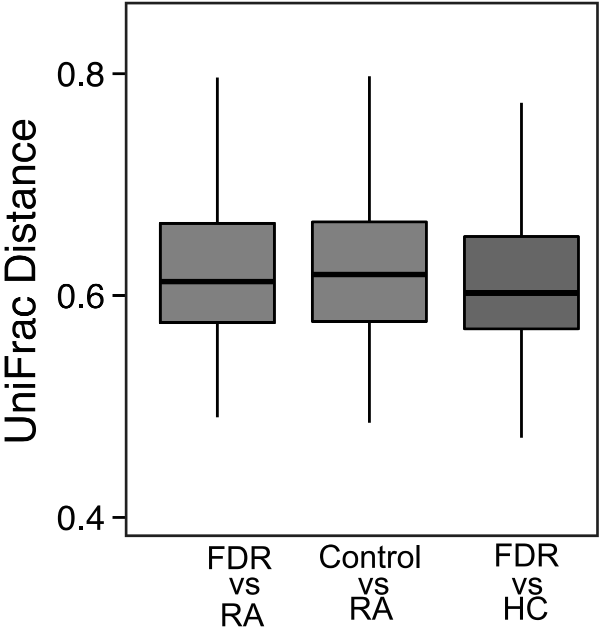

Figure S4. Boxplots comparing the inter-group UniFrac distances (RA vs FDR, RA vs HC and RA vs HC). The distances between FDR and HC are smaller than those between RA and FDR or HC, indicating disease may have stron-ger effects on the gut microbiota than genetic or environmental factors.

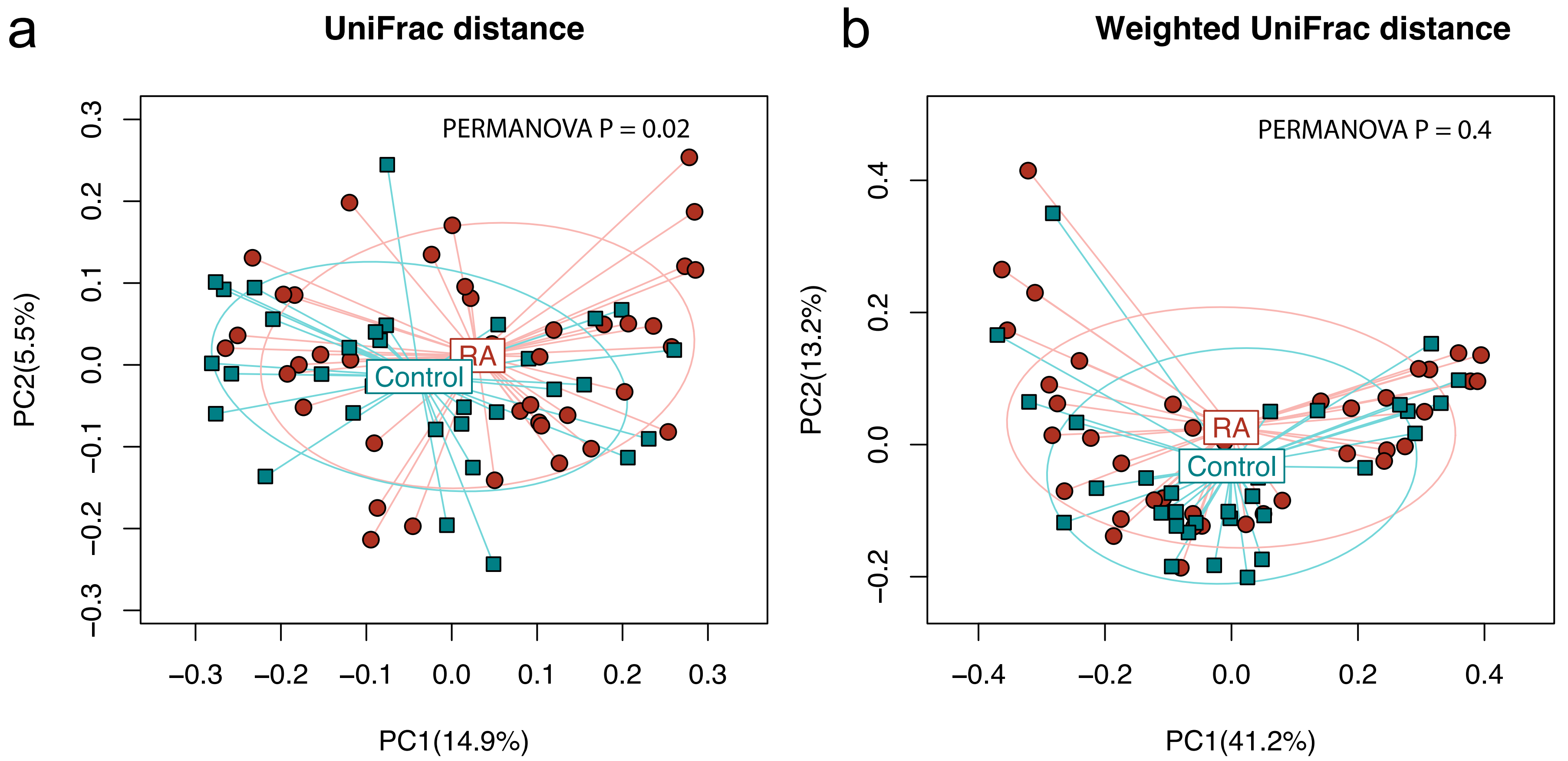

Figure S5. Principal coordinate plots based on unweighted and weighted UniFrac distances. PERMANOVA analysis shows the signifi-cant difference between RA and controls on unweighted UniFrac (A) instead of weighted UniFrac (B), indicating the microbiota change in RA mostly occurs in these rare and less abundant lineages. The percentage of variability explained by the corresponding coordinate is indicated on the axis. Each point represents a sample with red and blue color indicating the RA and control group respectively. The lines connecting to the centroid and the ellipses do not represent any statistical significance but rather serve a visual guide to group differences.

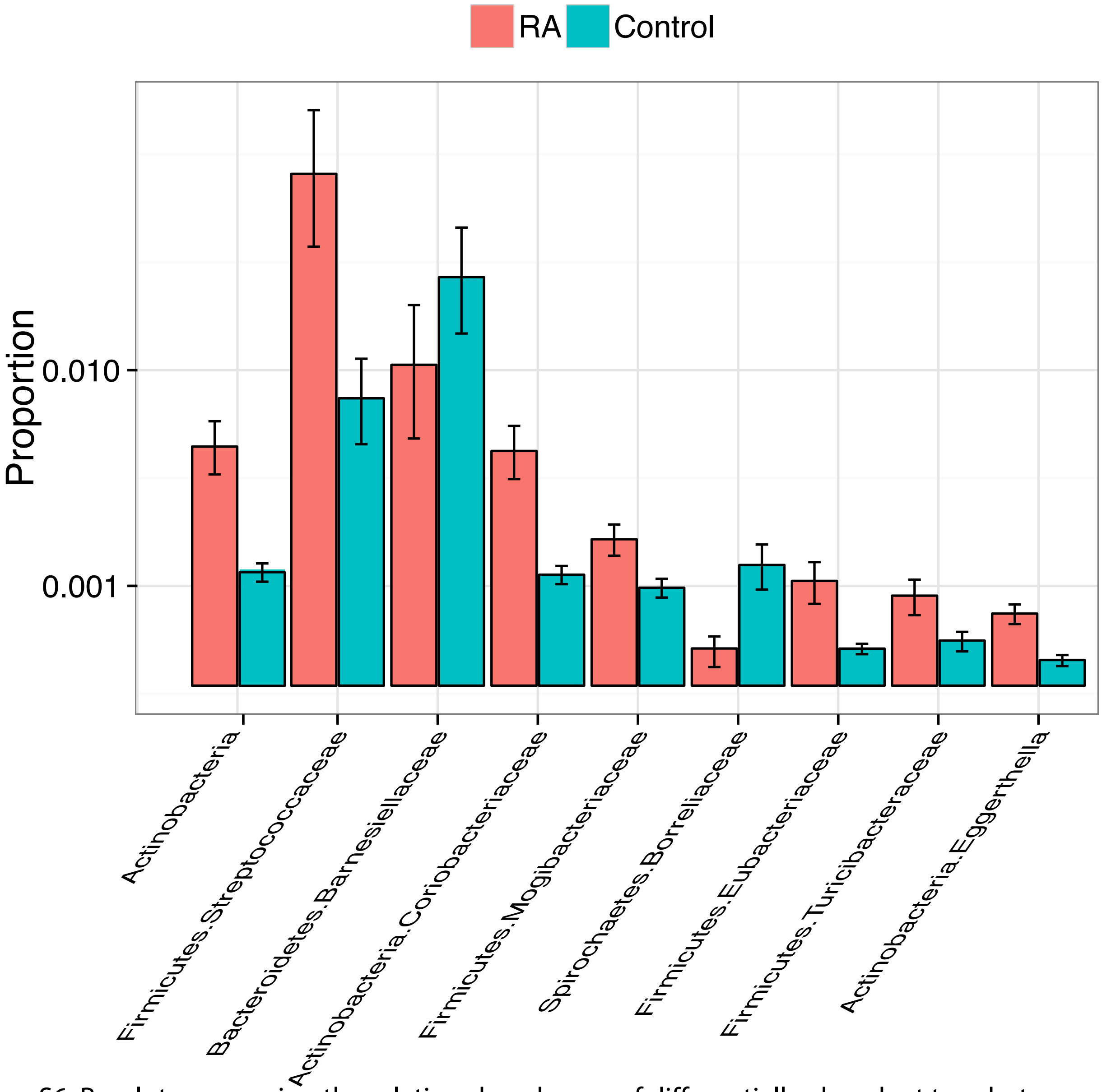

Figure S6. Barplots comparing the relative abundances of differentially abundant taxa between RA and controls selected based on an FDR of 15%. Error bars represents the standard error of the mean. The y-axis is on squared-root scale.

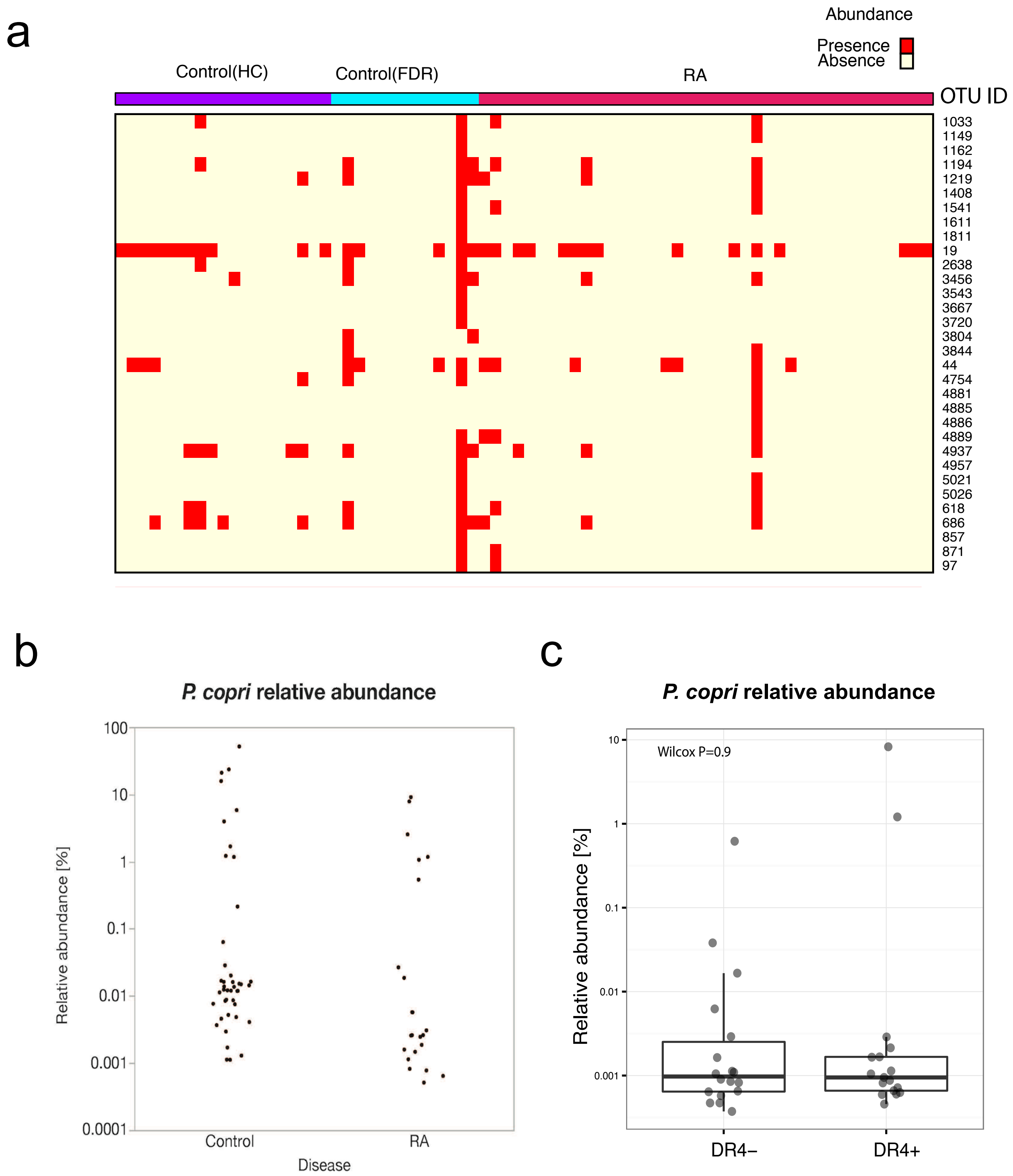

Figure S7. The relative abundance of Prevotella Copri (P. Copri) does not show significant difference between RA and controls. A. The presence of P. Copri OTUs is similar between RA and controls. The row names of the heatmap are IDs of these P. Copri OTUs. Column names are sample IDs. Red and yellow indicate presence and absence of the OTUs. B. The relative abundance of the P. Copri OTUs does not increase in RA patients. B. The relative abundance of the P. Copri OTUs in RA patients does not differ according to the HLADR4 status.

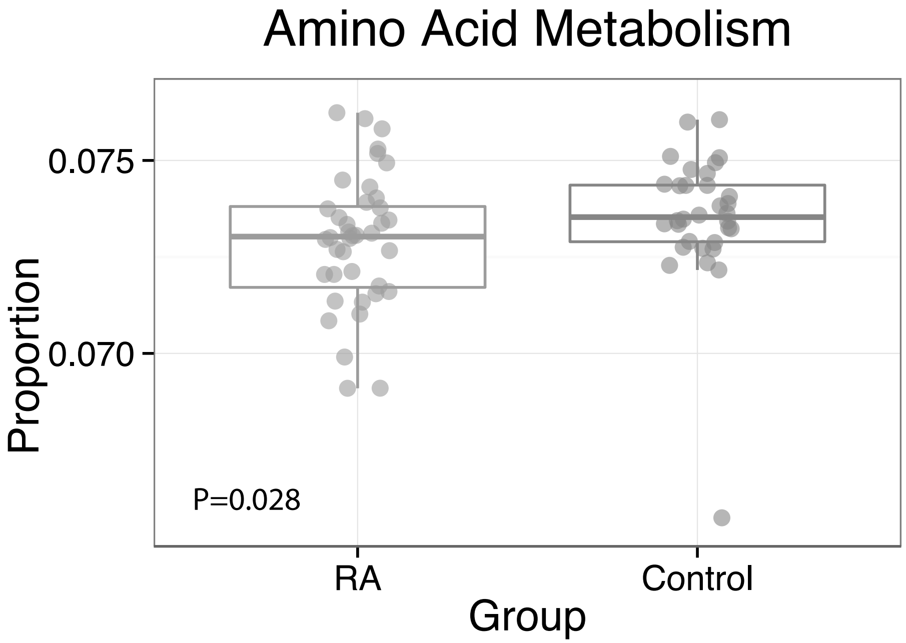

Figure S8. RA gut microbiota has decreased function in amino acid metabolism. The abundance of the KEGG pathway categories was calculated based on PICRUSt. The three horizontal lines of the box represents the first, second (median) and third quartile respectively with the whisk extending to 1.5 inter-quartile range (IQR).

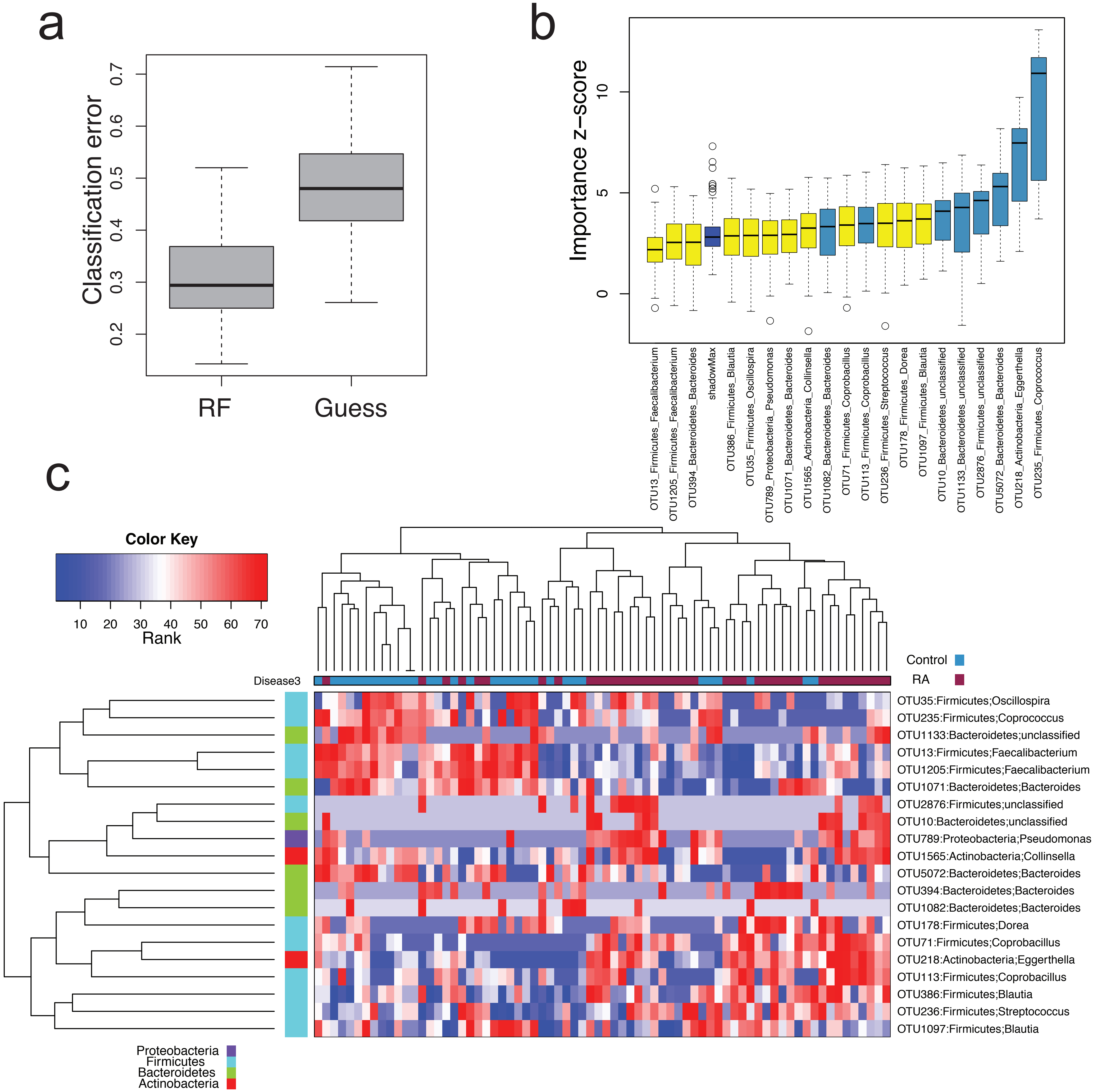

Figure S9. The predictive power of gut microbiota profile (species) for RA status assessed by machine learning algorithm random forests. Random forests, an ensemble classifier built upon many decision trees, was used to build a prediction model based on the OTU-level relative abundances. A. Comparison of the classification error of the random forests-trained model to guess, which always predicts the class label based on the majority class in the training data set. The boxplots are based on the results from 200 boot-strap samples. Random forests achieves significantly lower classification error. B. Predictive power of individual OTUs assessed by Boruta feature selection algorithm. Deep blue box-plots correspond to maximum importance Z score of shadow genera, which are shuffled version of real genera introduced to random forests classifier and provide a benchmark to detect truly predictive genera. Yellow and light blue colors show the tentative and confirmed genera by Boruta selection. C. Heatmap based on the abundance ranks of the Boruta confirmed OTUs. Red and blue indicates high and low abundance respectively. Hierarchical clustering (Euclidean distance, complete linkage) shows that RA samples tend to cluster together.

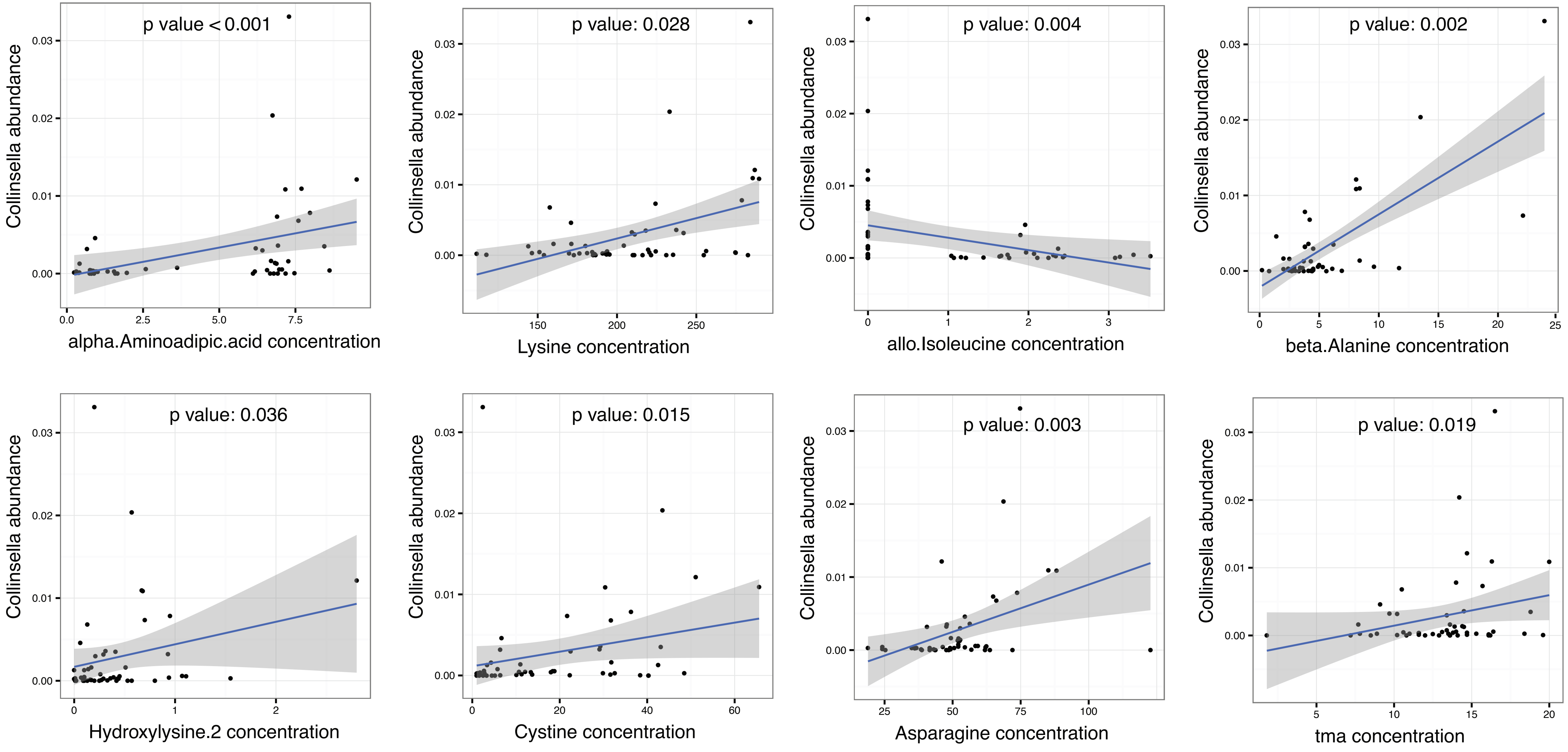

Figure S10. Scatterplots showing the correlation of the abundance of differentially abundant metabolite with Collinsella abundance. Significance was assessed by Spearman rank correlation test. The blue line shows the fitted linear regression line with the gray area indicating the 95% confidence band. P values are not corrected for multiple testing.

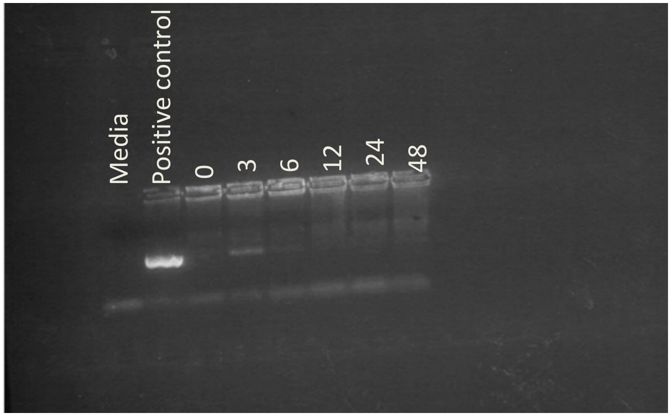

Figure S11 Collinsella does not colonize the gut.   Fecal pellets collected before mice were gavaged with *C. aerofaciens* and at various time points (3, 6, 12, 24 and 48 hrs.) after gavage were collected and PCR was done to determine the presence of the microbe. DNA from *C. aerofaciens* was used as a positive control and culture media as a negative control. After 12 hrs. of gavage, a very faint band was observed and 24 hrs., *C. aerofaciens* was not detectable in fecal pellets suggesting the microbe did not colonize the intestine.

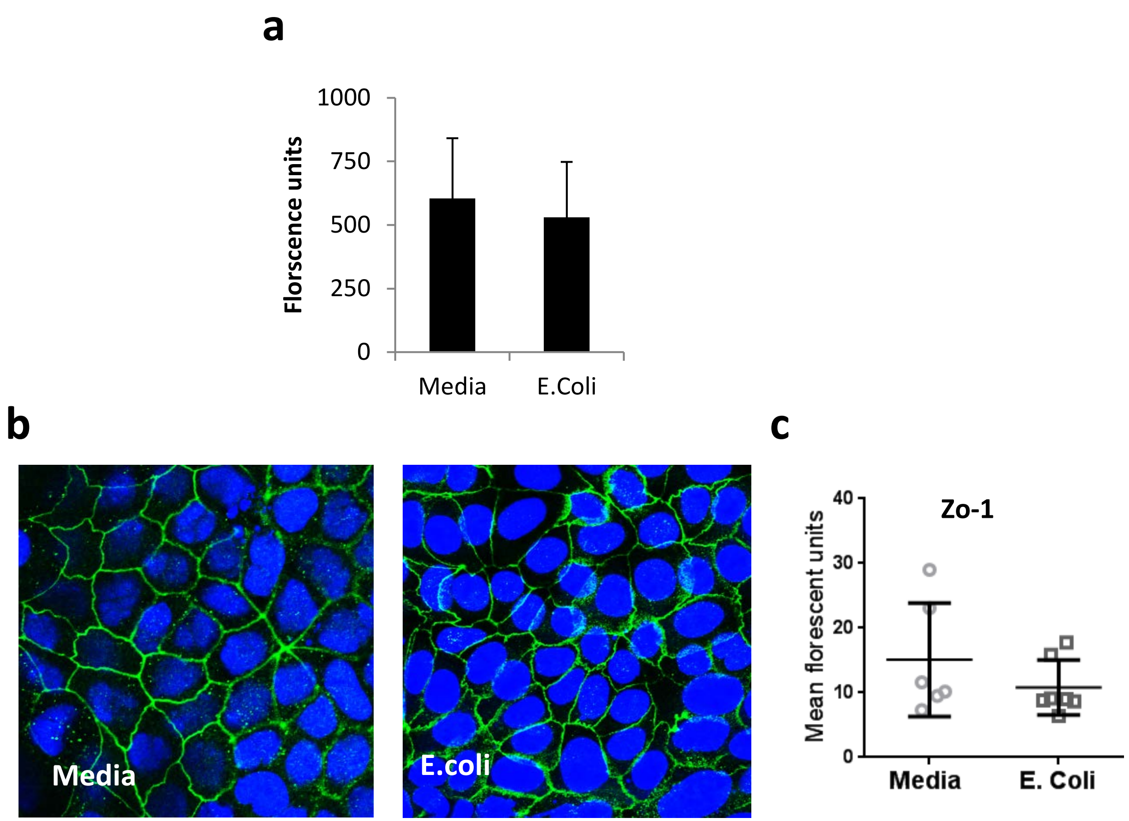

Figure S12. Non pathogenic E coli was used as a control microbe for gut permeability. A) Gut permeability in DQ8 mice administered E.coli or media did not show significant change. Sera of mice were tested for FITC-Dextran before and after treating mice with E.coli (10^8) for 3 weeks, P=NS (N=6 mice/group. B) Caco-2 cells were cultured with E. Coli similar to C. aerofaciens. CACO-2 cells cultured with or without E.coli stained with ZO-1 showed no significant difference in the expression of tight junction protein. C) Quantification of the mean fluorescence intensity of ZO-1 expression in CACO-2 cells cultured in the presence of E.coli or media, P=NS.
